# COVID-19 with peripartum cardiomyopathy: a case report

**DOI:** 10.1186/s42077-021-00204-z

**Published:** 2022-01-20

**Authors:** Sunaina Tejpal Karna, Pooja Thaware, Pooja Singh

**Affiliations:** Department of Anesthesiology and Critical Care, All India Institute of Medical Sciences, Bhopal, Habibganj, Bhopal, Madhya Pradesh 462020 India

**Keywords:** COVID-19, Cardiomyopathy, Congestive cardiac failure, Echocardiography

## Abstract

**Background:**

The world has been facing the novel coronavirus SARS-CoV-2 pandemic. The novel coronavirus primarily affects the lungs but also affects multiple organ systems including the cardiovascular system causing myocarditis, cardiomyopathy, and arrhythmias. Cardiomyopathy has been reported in patients with COVID-19; however, prognosis of peripartum cardiomyopathy in a patient with COVID-19 is still unexplored. More knowledge is required to understand the incidence of cardiomyopathy due to novel coronavirus SARS-CoV-2.

**Case presentation:**

We report a case of peripartum cardiomyopathy gravida 2 parity 2 COVID-19 confirmed patient who underwent an emergency preterm lower segment caesarean section (LSCS) for severe pre-eclampsia and intra-uterine growth retardation (IUGR) and landed up in acute congestive cardiac failure with pulmonary oedema. A postpartum 32 years female presented to our institute, a dedicated COVID-19 hospital with tachycardia, hypertension, anasarca, tachypnea with desaturation on room air. She had undergone emergency caesarean section for severe preeclampsia with intrauterine growth retardation. On post-operative day 2 (POD2), she complained of shortness of breath. On POD 3 she tested positive RT-PCR for COVID-19 infection. She responded to treatment with steroids. However, on POD6, She developed severe pulmonary oedema with poor ejection fraction necessitating endotracheal intubation and pressure control ventilation. Congestive cardiac failure was managed with diuretics and digoxin. Gradually oxygenation improved. She was electively ventilated for 3 days. Gradually, ejection fraction improved with the resolution of B lines. On the 9th POD, after a successful spontaneous breathing trial, she was extubated and non-invasive ventilation with bi-level positive airway pressure was attached. The patient was gradually tapered off of the non-invasive ventilation over 2 days. On the 11th post-operative day, she was maintaining oxygen saturation on nasal prongs and was sent to the ward.

**Conclusions:**

We recommend early use of bedside lung ultrasonography; echocardiography and close cardiovascular monitoring in severe COVID-19 infected pregnant patients who present with shortness of breath, tachypnea, and hypertensive disorders of pregnancy and previous cardiac abnormalities for expedite management and improved prognosis. An ideal case scenario for extubation may not be present, non-invasive ventilation with bi-level positive airway pressure post-extubation helps in patients with peripartum cardiomyopathy.

## Background

The current novel coronavirus SARS-CoV-2 pandemic has affected millions of people worldwide and numbers are continuously increasing. It is a deadly virus particularly deadly in the vulnerable and high-risk population. Such a high-risk group is pregnant females. Physiological changes in pregnancy such as reduced functional residual volume, altered cell immunity, and elevation of diaphragm increase the susceptibility of contracting COVID-19 infection. We are in a continuous state of learning about the COVID-19 disease its pathophysiology, complications and management. Cardiomyopathy in pregnant and non-pregnant patients is one such dreaded complication reported due to SARS-CoV-2 infection which can be proved fatal if not diagnosed during the early stages. Peripartum cardiomyopathy presents with typical signs and symptoms of acute congestive heart failure and symptoms may mimic physiological changes of pregnancy thus echocardiography helps confirm the diagnosis. Once diagnosed, management includes non-invasive or invasive ventilatory support, the institute’s standard protocol for the treatment of COVID-19, classical treatment goals of heart failure, and should include thromboembolic prophylaxis. Here we present a case where a gravida 2 para 2 confirmed case of COVID-19 underwent emergency preterm lower segment caesarean section (LSCS) for severe pre-eclampsia and intra-uterine growth retardation (IUGR) and landed up in acute congestive heart failure with pulmonary oedema and its successful management.

## Case presentation

A postpartum32years female presented to our institute, a dedicated COVID-19 hospital with tachycardia (heart rate of 110 bpm), hypertension (BP 180/110 mmHg), anasarca, tachypnea with desaturation on room air (SpO_2_ 84%). She had undergone emergency caesarean section for severe preeclampsia with intrauterine growth retardation at 30 weeks of gestation under regional anaesthesia three days back. She was transfused 1 unit of packed red blood cells on the first postoperative day (POD1). However, on POD2 she complained of shortness of breath with uneasiness and was managed with oxygen supplementation and Pritchard regime for the control of preeclampsia. By Pritchard regimen, a loading dose of 4 g of 20% magnesium sulphate IV was given, immediately followed by 10 g of 50% magnesium sulphate intramuscularly (5 g in each buttock) and maintenance dose 5 g of 50% MgSO4 IM 4 hourly in alternate buttocks for 24 h.

When there was no improvement, she was referred to our institute with suspicion of COVID-19 infection. The patient had no known contact with COVID-19 cases and had no travelling history. She is a known hypothyroid on tablet levo-thyroxine 50 μg once a day. There was no significant medical, family, psycho-social history, and surgical history.

She was initially managed for hypertensive crisis with severe preeclampsia with supplemental oxygen with high flow nasal cannula, tablet torsemide 10 mg 12 hourly, infusion labetalol at 10 mg/h, and magnesium sulphate for 24 h for seizure prophylaxis along with antibiotics and levothyroxine 50 μg OD as she was a known hypothyroid patient. However, with a positive RT-PCR for COVID infection, she was further given dexamethasone 8 mg 12 hourly for cytokine storm and therapeutic dose of subcutaneous enoxaparin 60 mg BD with Tab ecosprin 75 mg OD to prevent thromboembolic complications. Care was taken to avoid any further source of sepsis and intramuscular injections. She responded to treatment with decrease in oedema, controlled blood pressure, and saturation of 97% on room air and was shifted to the ward after 2 days of observation.

**Fig. 1:**
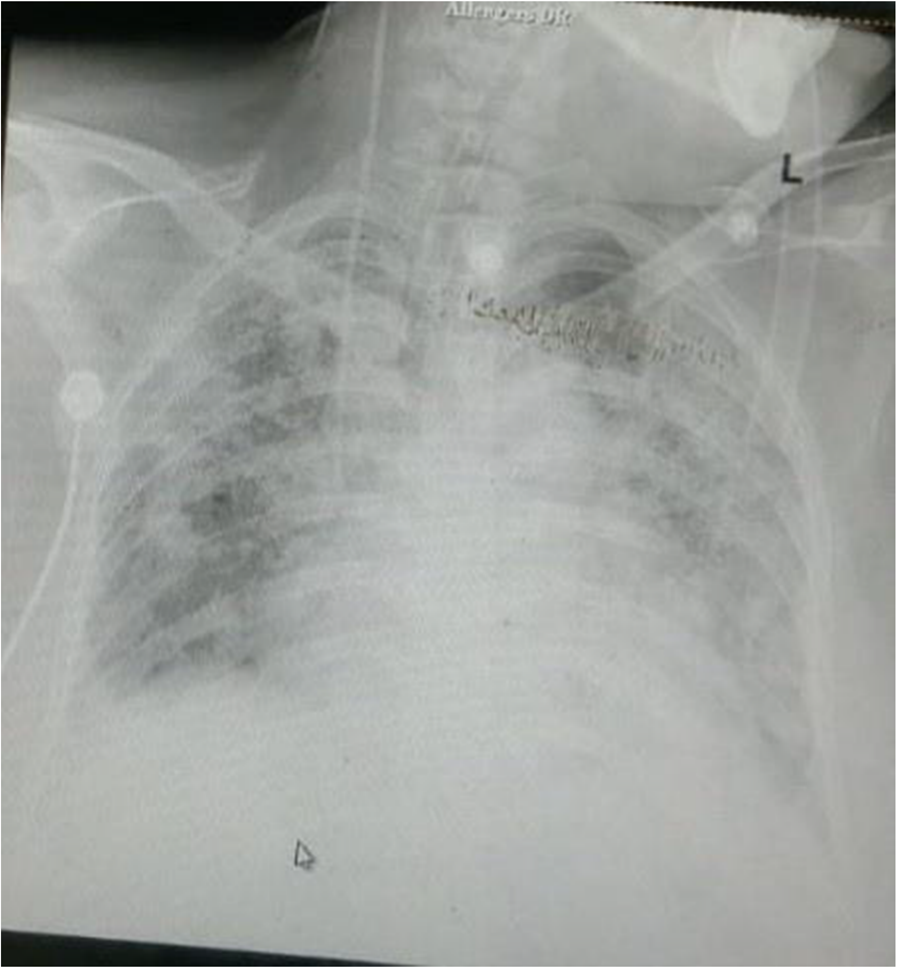
Chest X-ray on POD6 showing acute congestive cardiac failure with ARDS

**Fig. 2:**
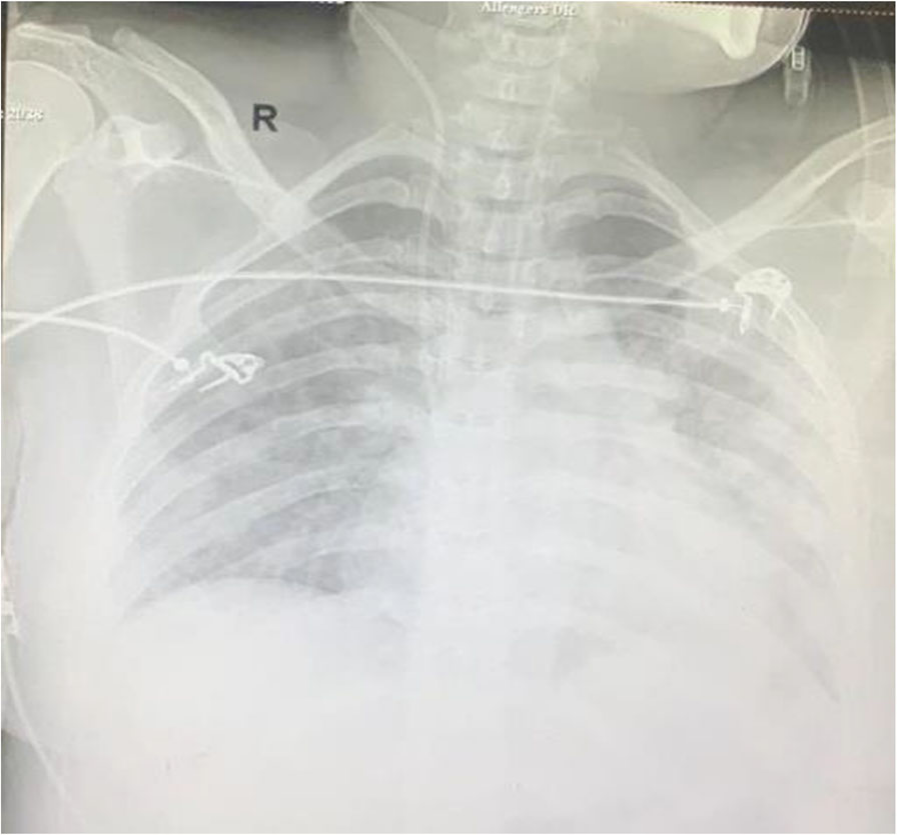
Chest X-ray on POD9

However, on the sixth postoperative day, she developed obtundation, severe pulmonary oedema (B-lines on USG Lung) with poor ejection fraction necessitating endotracheal intubation and pressure control ventilation with inspiratory pressure support (PS) of 22 mmHg and positive end-expiratory pressure (PEEP) of 10 with fraction of inspired oxygen (FiO2) 1.0. Her oxygen saturation remained 85–90% for the next hour (Table 1). Lung ultrasonography revealed bilateral B-lines, with poor left ventricular ejection fraction of 30%. She was diagnosed with acute congestive cardiac failure (CCF) with COVID pneumonia (Fig. 1). Management of CCF was done with furosemide infusion (20 mg/h) and intravenous digoxin 0.125 mg 12 hourly. Gradually oxygenation improved with FiO2 requirement decreased to 0.6. Antibiotics were empirically upgraded to meropenem with teicoplanin. A catheter was inserted in the right internal jugular vein for giving ionotopes, vasodilators, drugs, and to have access to blood samples. Arterial cannula was inserted in the right radial artery for the beat to beat blood pressure monitoring and drawing sample for arterial blood gas analysis. However, the next day, in view of hypokalaemia, digoxin was withheld with the initiation of nitroglycerine infusion (1 μg/kg/min), dobutamine infusion (5 μg/kg/min) and correction of hypokalaemia (Table 2). She was electively ventilated for 3 days. Gradually, ejection fraction improved to 50% with resolution of B lines and improvement in oxygenation. On the 9th POD, after a successful spontaneous breathing trial on pressure support of 10 mmHg, PEEP 5 mmHg, the trachea was extubated and non-invasive ventilation with bi-level positive airway pressure (BiPAP) attached with the same settings (Fig. 2). Immediately post-extubation, nitroglycerine infusion was gradually tapered off. Tablet carvedilol 3.125 mg OD and ramipril 2.5 mg BD were started to decrease afterload and improve left ventricular ejection fraction with continuation of diuretics (furosemide 20 mg IV 8 hourly) to decrease preload. On the 11th post-operative day, she was maintaining oxygen saturation 97–98% on nasal cannula at 2 L/min and was sent to the ward. The baby of the patient has tested negative for RT-PCR for COVID-19 infection.

**Table 1:** Serial arterial blood gas analysis and trend of prognostic markers

Arterial blood gas (ABG) and inflammatory and prognostic markers	POD3 On admission to our institute, Hudson’s mask 6 L/min	POD6 Before intubation on high flow nasal cannula FIO2 90%	POD7 Pressure control ventilation FIO2 40% PEEP 10 mmHg	POD8 Spontaneous breathing trial Pressure control PEEP 8 mmHg FIO2 40%	POD9 Spontaneous breathing trial Pressure control PEEP 5 mmHg FIO2 40% Before extubation	POD10 Post extubation on NIV BIPAP PEEP 5 mmHg FIO2 40%	POD11 Nasal cannula at 2 L/min
pH	7.442	7.412	7.491	7.460	7.486	7.473	7.492
P/F ratio	132	61	180	205	255	347	308
PCO2 (mmHg)	37	25	43.9	49.4	48	39.7	31.2
Neutrophil to lymphocyte ratio (NLR)	3	15.3	7.9	10.5	8.7	7.16	5.2
C reactive protein (mg/dL)	90	200			60		
LDH (U/L)			799	769			330
D-dimer (μg/ml)		3.62

**Table 2:** Laboratory investigations

Laboratory investigations	Pre-operative	POD1	POD3 On admission to our institute	POD4	POD7 Pressure control ventilation	POD8 Pressure support ventilation PEEP 8 mmHg FIO2 40%	POD9 Pressure support ventilation	POD10 Post-extubation on non-invasive ventilation
Haemoglobin g/dL	12.0	8.1	8.2	9.3	8.2	8.4	8.9	9.2
TLC/cumm	12,500	13,100	17.4	15.95	11.08	14.85	11.96	11.40
DLC Neutrophil %	72	78	94	92	87	84	87	86
DLC Lymphocyte %	23	26	05	06	11	08	10	12
Platelet count (Lac/cumm)	1.65	1.41	1.33	1.72	1.50	2.16	2.58	2.24
Urine albumin	Present ++	Absent						
Serum potassium (mEq/L)		3.92	3.2	2.76	2.91	3.8	3.9	3.8
Prothrombin time/INR			19.8/1.49			13.9/1.02		19.9/1.14
APTT (seconds)			25.9			40.6		45.3

The patient has been asked to follow up in the cardiology outpatient department after 6 months. At the time of submission of this case report, the patient has been discharged to home after testing negative for RT-PCR for COVID-19 infection. The patient was satisfied with the treatment and was very thankful to all the doctors, nursing staff and all the staff involved in her treatment.

## Discussion

SARS-CoV-2 is not just a respiratory virus, but it has multi-organ affinity (Spuntarelli et al., 2020)..Cardiomyopathy has an incidence of 33% in critically ill non-pregnant patients with COVID-19 (Guo et al., 2020). Various mechanisms proposed include increased inflammatory response, downregulation of angiotensin-converting enzyme 2 receptors, autonomic tone disturbance, increased endogenous catecholamines, hypercoagulable state, hypoxaemia, cardiopulmonary deconditioning, and peripheral deconditioning. All these lead to myocardial injury and conduction system damage which leads to hypotension, decompensated heart failure, acute coronary syndrome, tachyarrhythmia, bradyarrhythmia, and sudden cardiac death (Kochi et al., 2020).

A recently published case series described two cases of COVID-19–related cardiomyopathy in the pregnant patient. However, both of these patients possessed multiple risk factors for cardiac disease, and it remained unclear as to whether cardiomyopathy occurred as a direct complication of COVID-19, or secondary to multiorgan dysfunction (Juusela et al., 2020). Dilemma of diagnosis remains between peripartum cardiomyopathy (PPCM) and COVID-19-related cardiomyopathy.

In our patient, the differential diagnoses made were PPCM, hypertensive congestive cardiac failure due to severe preeclampsia, COVID-19-related cardiomyopathy, or myocarditis due to other cardiotropic viruses. PPCM is a rare acute life-threatening complication of pregnancy, is a form of dilated cardiomyopathy defined by left ventricular systolic dysfunction and cardiac failure in the last month of pregnancy or within 5 months of delivery, absence of previous heart disease, and absence of identifiable causes of cardiac failure. Echocardiogram in our patient showed moderate to severe left ventricular systolic impairment with an ejection fraction 35–40%. The left atrium and valves were normal. The structural changes within the myocardium secondary to hypertension causing impaired myocardial relaxation resulting in diastolic failure were absent in our case. The patient was found negative for the screen of cardiotropic viruses.

Our institute is a dedicated COVID facility. It provides free of cost investigations and treatment for COVID positive patients. With logistic issues and limited resources in face of the overwhelming COVID-19 pandemic, we were unable to get patient’s, serial cardiac biomarkers, cardiac magnetic resonance imaging, cardiac biopsy, high resolution computed tomography scan chest to establish COVID-19 as the direct cause of cardiomyopathy. Arcari et al. in their study have found that the cardiac biomarkers elevation was common in COVID-19 pneumonia and is associated with worse prognosis (Arcari et al., 2020). However, as per the disease course, the aetiology of cardiac failure in our patient is likely to have been a congestive cardiac failure with pulmonary oedema due to peripartum cardiomyopathy which may have been exacerbated by COVID-19.

The management of all peripartum cardiomyopathy is multimodal with diuretics, digoxin, dobutamine and nitroglycerine to decrease preload, improve cardiac contractility and decrease afterload. Hence, in the setting of COVID-19, another crucial aspect becomes the management of the inflammatory response and prevention of thromboembolic complications. We were able to manage the inflammatory response with dexamethasone 8 mg IV BD with therapeutic dose of enoxaparin. An ideal extubation scenario may not be available in patients with COVID-19. It is imperative to avoid iatrogenic ventilator-associated pneumonia. We extubated our patient after 48 h with a pressure support requirement of 10 mm Hg, PEEP 5 mmHg, and supplemented with BiPAP which was tolerated well by the patient.

## Conclusions

Due to lack of data on COVID-19 infected pregnant patients and cardiomyopathy, further studies on pregnant patients infected with COVID-19 are required. Ethical dilemma rules out any prospective or randomized controlled studies. Till the time such data is available, every case report may contribute to further learning in this vulnerable group of population if inflicted with COVID-19. We recommend echocardiography and close cardiovascular monitoring in severe COVID-19 infected pregnant patients who presents with respiratory distress and preeclampsia for timely management and prevention of fatal outcome. Management of COVID-19 inflammatory state should go alongside treatment of cardiac failure. Early extubation may reduce iatrogenic ventilator-induced injury.

## Data Availability

Not applicable

## Abbreviations

LSCS: Lower segment caesarean section
IUGR: Intra-uterine growth retardation
CCF: Congestive cardiac failure
PPCM: Peripartum cardiomyopathy
POD: Post-operative day
PEEP: Positive end-expiratory pressure
BiPAP: Bi-level positive airway pressure
